# Specificity and integration of meaning in self-defining memories of breast cancer survivors: clinical reflections to promote a narrative identity integration

**DOI:** 10.3389/fpsyg.2024.1433266

**Published:** 2024-08-14

**Authors:** Maria Luisa Martino, Valeria Sebri, Jefferson Singer, Katie Madow, Alice Viola Giudice, Davide Mazzoni, Maria Francesca Freda, Gabriella Pravettoni

**Affiliations:** ^1^Department of Humanities, Federico II University, Naples, Italy; ^2^Applied Research Division for Cognitive and Psychological Science, IEO European, Institute of Oncology IRCCS, Milan, Italy; ^3^Department of Psychology, Connecticut College, New London, CT, United States; ^4^Department of Oncology and Hemato-Oncology, University of Milan, Milan, Italy

**Keywords:** autobiographical memory, self-defining, breast cancer, long survivorship, body image, narrative integration

## Abstract

Potential traumatic events, such as breast cancer, can influence autobiographical memory (AM), interrupting the continuity of narrative identity. AM is based on a hierarchical search across different levels of specificity that are indexed from top to bottom when a memory is retrieved. In the breast cancer field, non-specific AMs are an observed clinical phenomenon. In particular, breast cancer survivors report issues related to self-defining memories (SDMs), specific and significant AMs that evoke strong emotions and sensory details at the time of memory. SDMs are linked to life goals and facilitate adaptation to critical experiences, preserving the continuity of identity. This study explored the narrative identity integration process of breast cancer survivors, analyzing themes, specificity, and integrative meaning in SDMs. Ten women participated in an online group support program centered on the integration of AMs linked to the cancer journey. Participants were asked to assess their body image perceptions, filling out an online survey three times, in which they had to report three SDMs each time. A reflexive thematic analysis of the SDMs identified three main themes: the onset of breast cancer; the labeling of negative emotions, and changes in the body. The results indicated inhibited retrieval of specific episodes, fostering a progressive failure in memory characterization and the concurrent meaning-making process. Participants struggled with connecting the memories to insights regarding their self and life, as well as relating the memories to external conditions and other individuals. Further studies might examine the impact of these difficulties on the psychological adjustment of BC long-term survivors. They could also explore cognitive reconstruction by reframing the memories and re-evaluating their traumatic meanings.

## Introduction

### Autobiographical memory functioning and self-defining memory in breast cancer experience

Within a narrative and constructivist perspective (Brockmeier, 2000; Angus and McLeod, 2004; Janoff-Bulman, 2004; Hermans and Dimaggio, 2004; Joseph and Linley, 2005; Neimeyer, 2006; Neimeyer et al., 2010), breast cancer is considered as a potentially traumatic experience by interrupting the continuity of the narrative self (Palmer et al., 2004; Cordova et al., 2007; Mehnert and Koch, 2007; Romeo et al., 2019; Martino et al., 2022). The traumatic condition is due to the sudden and unexpected alteration/shattering of basic elements governing the predictable relation between women and the external world (Janoff-Bulman, 2004; Joseph and Linley, 2005) and the rupture of the temporal continuity resulting in a crisis of meaning-making that supports the self-narrative of life interrupting self-continuity over time (Brockmeier, 2000; Neimeyer, 2004; McAdams, 2008; Martino and Freda, 2016). The crisis affects the meaning-making processes that support the individual’s personal life story and continuity of life, leading to a renewed quest for meaning under the changed circumstances (Davis, 2008; Freda and Martino, 2015; Kowalska et al., 2022; Park, 2022).

Accordingly, the diagnosis and treatment of cancer can destroy their daily life, the articulation of the past, present, and future, and their own visions of the world (Goula et al., 2020). The difficulty of psychological adaptation is linked to the risk of major traumatic outcomes derived from a stronger fear of recurrence and overall health anxiety (Cordova et al., 2007; Simard and Savard, 2009; Jones et al., 2014). Another relevant aspect links the psychological impact of the illness to the specific characteristics of cancer as a stressor: the intangible and internal nature of the threat, the uncertainty about the disease outcome, its unpredictable trajectories, and the threat’s chronological aspects, which prolong the criticality over time, and/or extend the different phases of the medical process (Martino et al., 2019).

Regarding the specific focus of this study, breast cancer influences the functioning and the production of autobiographical memory (AM) (Bergouignan et al., 2011; Rodrigues et al., 2023). Autobiographical memory refers to a knowledge base of personal information that includes specific episodic memories of past events and conceptual self-information (Conway and Pleydell-Pearce, 2000). AM relies on a hierarchical retrieval process across different levels of specificity that is accessed from top to bottom when the subject searches for a memory. Autobiographical knowledge can be aligned in general phases or stages of life—“life periods”; in more limited and thematic timelines composed of months, weeks, or days—“general events”; or it may possess specific imagistic qualities linked to a given moment in time—“event-specific knowledge.” AMs are accessed first through abstract categories, and greater effort is required to reach specific details; a working memory deficit (e.g., traumatic experiences) may impair retrieval and lead to overgeneralization and a lack of specificity in AMs (Williams et al., 2007). Difficulties in retrieval AM, as in the case of overgeneral memory, are due to its association with some types of psychopathology, such as depressive disorders (Hallford et al., 2021) or post-traumatic stress disorder (Dalgleish et al., 2008; Ono et al., 2016; Barry et al., 2018). Anxiety can also interact with AM retrieval, leading to fewer emotional details in AMs (Morel et al., 2015). Furthermore, optimal and redemptive integration into the self of a negative experience in life is an outcome observed in post-illness patients with good adaptation (Martínez-Hernández and Ricarte, 2019) and a more general indicator of psychological adjustment (Blagov et al., 2022).

Self-defining memory (SDM) is a specific type of autobiographical memory, which plays a key role in the construction and integration of personal identity (Singer et al., 2007; Cuervo-Lombard et al., 2021). SDM is a particularly central and significant AM that evokes strong emotions and sensory details at the time of recall; it is characterized by vividness, emotional intensity, repeated recall, link to memories that share similar thematic content, and connection to ongoing concerns or conflicts in the personality (Singer and Bonalume, 2010). These memories have a particular thematic importance for self-understanding, becoming useful in communicating oneself to others. Literature has demonstrated a significant role of SDM in the construction of life goals and self-esteem and in adaptation and recovery from critical emotional experiences while preserving a coherent self-identity (Bluck, 2003; Singer and Blagov, 2004a; Liao et al., 2018). Optimal employment of SDM lies in individuals’ ability to derive an integrative meaning for oneself and one’s life from the memory, carrying out a “meaning-making” function for the self. This is an additional step of attributing meaning to one’s memories by relating them to lessons about themselves, important relationships, or life in general. In this way, self-defining memories allow the memory to influence the self, reinforcing its affective value and relationship to relevant life goals (Singer and Blagov, 2000; Singer and Blagov, 2004b; Blagov et al., 2022).

The diagnosis of breast cancer and related treatments usually remain strongly imprinted in the AM of individuals. Breast cancer survivors may experience AMs that are intrusive or have a high negative emotional impact related to illness, leading to a disconnection between some negative self-representations and a healthier holistic sense of self. Damage to the body due to oncological treatments generally leads to changes in body image and perception due to aesthetical differences, such as scars and weight (Sebri et al., 2022). Thus, the bodily self (defined by bodily changes and perceptions) and narrative self (that includes AMs) are dependent on each other, affecting individuals’ daily wellbeing (Sebri et al., 2021; Sebri and Pravettoni 2023). These assaults on the self by traumatic memory can have long-term effects on attitudes, behaviors, moods, and meaning-making of life events (Martino et al., 2023). Among different cancer experiences, a low specificity of AMs in patients with breast cancer is relevant (Giffard et al., 2013; Morel et al., 2015). Singer and Salovey (2014) suggested that overgeneral memory may be the repression or blocking of negative-toned information from consciousness. Furthermore, rumination, avoidance, and/or emotional suppression have been postulated as processes that, alone or in conjunction, underlie deficits in the retrieval of specific AM (Maccallum and Bryant, 2008; Sansom-Daly et al., 2016). Emotional avoidance or the level of anxiety may explain overgeneral memories and affect the capacity to retrieve more emotional details in cancer survivors’ memories (Sansom-Daly et al., 2018). On the contrary, individuals who show a strong tendency to draw integrative meaning or life lessons from their memory report high levels of adaptation and recovery from struggling emotional experiences (Singer and Blagov, 2000; Blagov and Singer, 2004; Singer and Conway, 2011; Blagov et al., 2022). To date, a small body of studies on the impact of cancer on memory and AM have been conducted, particularly in breast cancer survivors (e.g., Rodrigues et al., 2023), but few studies have focused on SDMs in subjects with cancer, yet these memories could be a valuable source of self-exploration and guidance in efforts to build resilience and integration in survivors (Nieto et al., 2019).

Starting from the aforementioned literature, this study aims to explore themes, specificity, and integrative meaning in SDMs linked to the experience of breast cancer in survivorship. It focused on long-term cancer survivors who participated in an online support group directed toward the adaptation and integration of body image and the re-evaluation of memories to enhance wellbeing and psychological health. This study allowed us to highlight preliminary clinical reflections that could contribute to focused interventions based on the reframing of AMs in breast cancer survivorship.

Some exploratory questions we considered were as follows: (1) How might body image themes be integrated into persisting SDMs linked to the breast cancer experience? (2) What forms of meaning-making with regard to self-understanding, relationship to illness, and experiences of social interaction might be present in the breast cancer-linked SDMs of these survivors? (3) How might the group intervention with its focus on the survivors’ SDMs affect the integrative meanings they assign to their breast cancer-related memories?

## Materials and methods

### Institutional review board statement

This study was performed in line with the principles of the Declaration of Helsinki and approved by the Ethics Committee of the University of BLINDED (n45/23, 18 April 2023).

### Participants

Twenty women with a breast cancer diagnosis in the past (7 years ago, on average) agreed to participate in a psychological research-intervention group focused on the management of self-defining memories in reference to cancer-related issues toward the body. However, two of them were undergoing chemotherapy during the project. Therefore, 18 women participated in the psychological intervention, which was carried out from April to June 2023. All participants were breast cancer survivors and met the inclusion criteria, as follows: (1) women who were 18 years and older (2) people with a diagnosis of breast cancer, and (3) the lack of current oncological treatment; women could have undergone a variety of past oncological treatments. Otherwise, exclusion criteria were related to cognitive impairment, inability to sign the informed consent, and/or mental disorders that prohibited their participation in the study (e.g., women who did not understand the study). A total of 14 women responded to open-ended questions both at the beginning and at the end of the psychological intervention and 1 month later (age range: 35–61; *M_age_* = 48.64; *SD_age_* = 7.78). The majority of participants had obtained a bachelor’s degree (63%), lived in the North of Italy (68%), and worked as white-collar employees (94%). In addition, nearly half had received individual psychological therapy in the past (47%), had a partner (58%), and had one or more children (68%).

### The bodily traumatic memory program (BTMP): a new group psychological intervention

The psychological intervention consisted of six online weekly sessions, each lasting 2 h. It was conducted by an expert psycho-oncologist with extensive professional experience. BI issues and the content, in general, were based on previous studies focused on body compassion (see Sebri et al., 2024, in submission). Drawing from psychological theories and validated group interventions using self-defining memories among different populations (Nieto et al., 2019; Raucher-Chéné et al., 2021), the main goal was to promote a positive BI after oncological treatments. The group played a significant role by promoting the integration and transformation of self-defining memories linked to the breast cancer journey (from diagnosis to treatment and the end of oncological interventions), leveraging the power of membership dynamics (see Sebri et al., 2024, in submission). Therefore, the psychological program explored and reframed self-defining memories connected to body parts, particularly those affected by breast cancer, increasing positive interoceptive sensations and the reappraisal of the overall relationship with the body, along with its related emotions and behaviors.

### Procedure

The invitation to participate in the online programs was posted on social networks and sent via a commercial mailing list. This way, breast cancer survivors had the possibility to express their interest in taking part in this study. This way, a self-selection method was applied to recruit participants who were interested in the present research-intervention group project. After receiving their application, the inclusion criteria were assessed.

Women who were involved in this study had to meet the following inclusion criteria:

1. 18 years and older,
2. having received a diagnosis of breast cancer in the past years (at least 5 years ago),
3. absence of oncological treatment ongoing,
4. understanding and speaking of Italian language.

Moreover, breast cancer survivors who showed physical or psychological impairments that prohibited their participation in the study, such as inability to understand the study or to sign the informed consent, were excluded from this research. They received an email with the information sheet and were briefed about the contents of the psychological interventions, how the sessions were organized, and the request to respond to three open questions at three different points in time. Moreover, they were asked to sign a written consent form.

Participants took part in six online psychological sessions in a group conducted by a psychologist with expertise in psycho-oncological issues.

### Tools

To focus on the SDMs, the women were asked to fill in a written form through a Qualtrics online survey. They were requested to retrieve three SDMs at three points in time:

- before participating in the group intervention (T0),
- at the end of the intervention (T1),
- after 1 month (T2).

We chose these three times to explore whether the SDMs showed changes in the quality or in the typology of memories between the times before and following the participation in the group intervention. In addition, we were interested in the maintenance of potential transformations (T2).

At the beginning of the group intervention, the characteristics of SDMs were shared with the women.

The proposed task, drawn from an adaptation of the task to retrieve SDMs in the manual of Singer and Blagov (2000–2001), was as follows (the same task for each detection):

*Each of you has had a specific and unique journey in your experience of illness and treatment for breast cancer. We ask you to go back in your mind, think about your illness and treatment journey that you went through, and write three memories of specific events, relating to the different phases of your experience, which have left a powerful impression “inside” you. Try to imagine wanting to tell your specific illness and treatment journey to a dear friend of yours, recalling in your memory the episodes and emotions that the experience evokes in you.*

*When writing the three specific memories, we ask you not to go beyond the lines provided and to provide a caption for each memory.*

### Method of data analysis

A multilevel qualitative procedure was adopted to analyze the women’s SDMs. First of all, the memory themes were coded, and, subsequently, the other SDM variables—specificity and integrative meaning—were coded within each time, T0-T1-T2.

(1) A reflexive thematic analysis (RTA) of memories was conducted through the methodology proposed by Braun and Clarke (2006) to explore themes and subthemes that emerged in the memory narratives.

The analysis was conducted by two researchers (M.L. and V.S.), carrying out a collaborative and reflective approach, which sought to highlight overarching themes that might express the central concerns of the survivors as they have moved through their cancer experience. When there were discrepancies in agreement between the first two authors, the third and fourth authors (J.S. and K.M.), who were not involved in the first phase of analysis, provided interpretations that allowed for all of the authors to reach consensus.

The analysis was conducted following the six-step process:

Phase 1: Familiarization and deep dive into the data and construction of preliminary notes on the data.
Phase 2: Generation of initial codes. The coding process was used to produce interpretative labels attributed to the portions of text that expressed thematic aspects relevant to the study’s focus on shifting relationships to body image.
Phase 3: Theme generation. The coded data were reviewed and analyzed in order to identify possible combinations according to shared meanings in order to build themes and subthemes. This involved merging multiple codes that shared a similar underlying concept or feature of the data into a single code.
Phase 4: Review of potential themes. This phase required the researchers to conduct a recursive review of themes in relation to the coded data elements and overall narrative material.
Phase 5: Definition and naming of the themes. In this phase, the final overarching themes and subthemes were defined that best encompassed the entire narrative material in relation to the perception of the body over the extent of the cancer experience.
Phase 6: A triangulated discussion of the results with the other authors (A.V. G.; D. M; M. F. F.; and G. P.) to confirm the comprehensiveness and meaningfulness of the thematic analysis.

(2) Analysis of the specificity and integration of meaning through the methodology proposed by Singer and Blagov (2000–2001) and Blagov and Singer (2004):

*Narratives of specific memories*. A specific memory narrative has at least one statement of a single event. A single event statement is a statement in which the attention is clearly focused on an event that meets the following criteria: (1) It is a single event. (2) It has a short duration, less than a day. Typically, specific memories consist of several interrelated statements of single events that chronicle a unique and brief uninterrupted sequence of perceptions and actions. The time and place are often specified. Often many details are provided, making it possible to imagine the setting and actors of that particular event. Participants are identified by names or other labels and described through their dialogue, emotional responses, actions, appearance, physical location, and other attributes. The specificity of the details varies from purely descriptive memories to reflective memories in which the remembered “steps out” of the narrative to provide contextual information and make inferences about the meaning of the event or the memory itself. Broader contextual information can present the event as embedded in a more general narrative beyond the time and place of the particular event.

*Narratives of Integrative memories* contain statements that give meaning to the memory described. This meaning is usually expressed in statements about what the memory has taught the individual (e.g., “the lesson learned” or “from that moment on I understood.”); these insights can be expressed about life in general or specifically about the individual’s life and sense of identity.

Following the analysis of memories according to the criteria of specificity and integration, they were categorized by assigning the following level scores and observing the distribution of percentage scores within each interview phase (T0-T1-T2) (Table 1).

**Table 1:** Categorizations of specificity and integration of meaning in SDMs.

4: Specific and integrative
3: Not specific and integrative
2: Specific and not integrative
1: Not specific and not integrative
0: No memory provided (only of a keyword, emotion, or sensation)

## Results

The first level of the results shows the main themes and subthemes that emerged from the qualitative analysis of the SDMs shared by women over the three times. Each memory had a minimum length of 1 line and a maximum of 24 lines. The whole memory narrative corpus was composed of approximately 6,000 words (Table 2).

**Table 2:** Main themes and subthemes of the whole corpus of SDMs over three times.

The onset of breast cancer	The labeling of negative emotions	Changes in the body
The sudden and unexpected irruption	Loneliness and isolation	Difficulties in recognizing the body
The cancer inscribed in the genes’ history: Between familiarity and repetition.	Fear and terror of death	The relevance of inner sensations
The subjective speculation about the causes of the cancer	The loss	The visibility of bodily changes
	The sense of emptiness	The body as a betrayer

The first level of results regarding themes that emerged in the women’s memories concerns **The Onset of Breast Cancer**. The memories in this main theme focus on the onset of cancer, the way in which the painful discovery occurred in her life, and their journey into the beginning of the treatment. This main theme is comprised of the following subthemes:

*The sudden and unexpected irruption*. This subtheme embraces memories of experience linked to the discovery of cancer vividly imprinted in one’s memories, often occurring by an accidental discovery, which leaves one disoriented and helpless in the face of the interruption of self-continuity.

*… in April 2017 while I was at work in front of the PC, I suddenly felt pain in my right breast and as soon as I touched myself I felt a hard bullet. For months I didn't say anything to anyone because having just had a gynecological examination I didn't think it could be anything serious. As the months passed, however, the anxiety grew because I subconsciously knew that the situation was abnormal. At the end of August I had a mammogram and within a few days I sank into oblivion … (Id 9, 61 years old, year of diagnosis 2014).*

*… At that moment I booked the ultrasound with a positive approach because it seemed paradoxical to me that it could happen at the same time as my sister; instead there was a nodule, small and 80% nothing serious but given my familiarity, they advised me to do a nodule aspiration. From that moment my life changed further.(Id 1, 40 years old, years of diagnosis 2016).*

*The cancer inscribed in the genes’ history*: Between familiarity and repetition. This subtheme encompasses memories connected to the discovery of cancer and the search for meaning linked to the internal question “why me?.” Often, this search is part of stories of familiarity/hereditary breast cancer in female family members which, as emerged in previous studies, provides a reassuring explanation of the events by placing them in a three-generational chain. On the other hand, it inserts women in a story destined to repeat itself within a script whose ending appears already written.

*… I immediately understood that it was bad also because in my family I had been burned when I was 16 by what had happened to my favorite aunt. She also had breast cancer and died after a 9-year ordeal. Since then I have always been on the alert and told my parents that I was afraid of getting cancer[…]I will always remember the look of the radiologist and the silence in the semi-dark room. Even now she makes me cry; I already knew it; I was sure, even before the news was bad. I don't know how I had this certainty… History proved me right. I discovered the familial mutation in me… I discovered the familial mutation in me and in other family members, actually the majority. It gave meaning to my aunt's death and feared that I would end up the same way. Even today this fear does not abandon me…(Id 2, 61 years old, year of diagnosis 2022).*

*The subjective speculation on the causes of the cancer*. This subtheme captures memories connected to the search for a sense of control of events by constructing subjective speculations, potential contributors, and predictability around the onset of the cancer.

*… The body rebels. I was going through a stressful period of work that was affecting my well-being, I couldn't manage the work pressures and role changes I was facing in the company and I didn't have a manager who could manage the staff correctly…(Id 7, 47 years old, year of diagnosis 2019).*

*… It had to arrive because my aunt had it too… I'd been on the alert all my life… so it arrived. I often said this to my mother…I felt it (Id 2, 61 years old, year of diagnosis 2022).*

The second main theme that emerged in the women’s memories concerns the **Labeling of Negative Emotions**, that is, putting into words and naming the emotions felt at the time of events. For the most, these are emotions experienced at the time of memory and then relived through the act of narrating the memory. This main theme comprises the following subthemes:

*Loneliness and isolation*. These SDMs frame and put into words the sense of emotional loneliness connected to not feeling fully understood by others while going through the breast cancer journey. They also depict the sense of isolation experienced before receiving the diagnosis and during some critical moments of the treatment process.

*…I did what I could but it's full inside, even the lymph nodes, before telling you what to do, you have to do a pet scan to rule out metastases… if you have relatives you contact them, as you can't do it alone… I didn't have any relatives, no one at that time … (Id 8, 47 years old, year of diagnosis 2021).*

*… I was lucky, what my mother often told me (wanting to encourage me) at the time still seemed to me to belittle what I was going through. I felt alone, even though I wasn't, maybe I didn't understand. It was July and everyone went to the beach. I suffered as if I could no longer hug the sea there … (Id 7, 47 years old, year of diagnosis 2019).*

*Fear and terror of death* emerged in memories, activated by the diagnosis of cancer within oneself and in the collective thought, opening up to catastrophic scenarios, for example, incessant thought metaphors

*… Then the most persistent feeling was a kind of bewilderment and the idea that “cancer is a journey where you know how you will end up, but not where you will end up.” Because in my life unfortunately I have more than one example of young women dying of cancer, all relapses … (Id 6, 35 years old, year of diagnosis, 2020).*

*… Once I switched to the radiotherapy I had some difficult moments and often when I took a shower I cried and let the tears flow with the water.(Id 2, 61 years old, year of diagnosis 2022).*

*The loss*. These SDMs are linked to the emotion of psychic and physical loss that the cancer has introduced into women’s lives, loss of one’s beauty and body image, loss of relationships, and loss of future motherhood.

*… I had my period; I just had had surgery, yet I underwent hormonal stimulation to have 1 chance of being a mother… the pain of not being able to have 10 eggs… hormone therapy and menopause at 39, the end of a destructive relationship 6 months earlier. I had nothing left… and today I have been closed and alone for years. I don't even have hope anymore… (Id 8, 47 years old, year of diagnosis 2021).*

*… I can't recognize myself and I get nervous. I'm disgusted. I hate myself because I'm so hungry … I will never be the same as before … (Id 1, 40 years old, years of diagnosis 2016).*

*The sense of emptiness*. These memories contain narratives that depict a sense of subtraction of physical and psychic energy caused in part by the care and treatment required. The women’s strength is spent in fighting one's ongoing battle, draining energy for reinvestment in future life.

*… When I finish the treatment and do the checks, which are currently going well, I feel lucky because I know it could have gone differently. But at the same time, I feel drained of all my energy and sometimes apathetic and tired. I feel as if my energy was lost in this journey, and today I am no longer able to be the same as before. I've tried, but sometimes I really struggle to wake up and smile…. (Id 2, 61 years old, year of diagnosis 2022).*

Finally, the third main theme in women’s memories concerns was **Changes in the Body**. Women mentioned about their bodily changes after oncological treatments, increasing negative perceptions of their own bodies. Particularly, five subthemes emerged by participants were as follows:

*Difficulty women faced in recognizing their bodies. This theme was due to actual physical changes, such as increased weight gain and menopause, which had a negative impact on their overall wellbeing.*

*…Surgery is nothing compared to follow-up care. Menopause induced at age 34 and unnatural. The binges on caloric food, the blocked metabolism and the 10 kg of weight have led to feelings of inadequacy … (Id 1, 40 years old, years of diagnosis 2016).*

*… From November 2020 to February 2023, 2 years have passed, and I gained between 7 and 10 g. I can't recognize myself and I get nervous. I'm disgusted. I hate myself because I'm so hungry, it's one of the few things that give me pleasure and doesn't make me think of negative things. I eat and I don't want to stop, but then I feel guilty for indulging in that tiramisu. This really cheers me up momentarily … (Id 4, 38 years old, year of diagnosis 2020).*

*Inner sensations*. Second, participants emphasized their tendency to focus on inner sensations, particularly pain. Consistently, physical pain could lead to discomfort in the body, with relevant consequences in daily life.

*… Finally it's my turn: I'm back in the operating room. In addition to having to enlarge the surgical area on the left side, there is also carcinoma on the right side and the axillary lymph nodes need to be removed. At that moment there was panic on my part. That was the only time I cried. On November 6th, I went back to the operating room for enlargement on the left and lymph nodes on the right. More weeks of pain, of not knowing what position to stay in bed to avoid feeling pain, more days of not being able to shower or wash myself fully …. (Id 2, 61 years old, year of diagnosis 2022).*

*Visibility of physical changes*. Moreover, participants highlighted their worries about showing bodily signs after surgery. Specifically, a woman expressed their concerns about the visibility of physical changes, and how others would notice them.

*… I would like a "silent" body. Unfortunately, things didn't go exactly as they should have. There was a problem with the prosthesis and I am currently waiting for a new surgery. My biggest concern is that my body "tells" what happened to me, that is, that a disharmony in my form is visible to everyone. Nonetheless, last summer, a few months after the surgery, I returned to the beach, in a bathing suit, and to this day I still regularly go swimming in the pool. Naturally, now that I know that another operation awaits me, the hope of achieving a better result is accompanied by the fear that this situation could arise again ….(Id 10, 42 years old, year of diagnosis 2018).*

*Body as a betrayer*. Fourth, the body is perceived as a betrayer due to the cancer onset. Accordingly, women experienced the fear of cancer recurrence, highlighting their worries about the possibility of a new oncological diagnosis.

*… January 2023 I finished hormone therapy and I should have felt free again, but in reality, compared to five years ago my relationship with the operated breast has not changed because I always feel different and with the fear that sooner or later the tumor will return … (Id 9, 61 years old, year of diagnosis 2014).*

*… I finished hormone therapy, but instead of feeling over the moon, I continue to be afraid that the disease will return. I still don't look at myself naked in the mirror and I try to touch my operated-on breasts as little as possible … (Id 12, 47 years old, year of diagnosis 2021).*

### Characteristics of SDMs—specificity and integration of meaning—within each time of detection

The second level of the results shows the distribution of percentages of the characteristics of SDMs in terms of specificity and integration of meaning within each time of detection.

Memories produced by 11 women at T0, before participating in the group intervention, show how, compared to 20% of memories not fully provided, 43% of the memories are characterized by an absence of specificity and integration of meaning, remaining on an overgeneral level (general event of a life period) which attests to difficulties in the process of retrieval and recollection of specific memories of one’s cancer journey:

*The importance of menstruactions. Surgery is nothing compared to follow-up care. Menopause induced at age 34 and unnatural. Caloric food binges, blocked metabolism, and the extra 10 kg resulted in feelings of inadequacy.(Id 1, 40 years old, years of diagnosis 2016).*

*The lightheartedness lost. When I stopped the hormone treatment, my body deflated, I regained fitness and improved it with physical activity and healthier eating habits. The lightheartedness, the lightness that on average a young woman feels I experience at times. (Id 1, 40 years old, years of diagnosis 2016).*

In total, 30% of the memories show characteristics of specificity but from which a process of integration of the meaning starting from the narrated memory, for the self and/or one's life, does not emerged.

*Before the vacation. I was alone, I had not wanted to postpone the judgment. I asked the doctor: but are you really sure? Couldn't it be a false positive? No, it does not, there is no doubt, surgery is needed, as soon as possible … (Id 3, 52 years old, year of diagnosis 2020).*

It seems interesting to highlight how 7% of memories, although they do not show specific characteristics, access a process of integration of negative and devaluing meaning-making for the self and one’s relationship with future life and with one’s world view.

*It's not me anymore. I keep telling myself how lucky I am, how good I am, how full my life can be, how many good things I could do, how grateful I should be… but in reality, everything is destroyed, I'm no longer me, I have nothing, nothing it makes more sense or value to me. I'm Surviving (Id 8, 47 years old, year of diagnosis 2021).*

**Figure 1:**
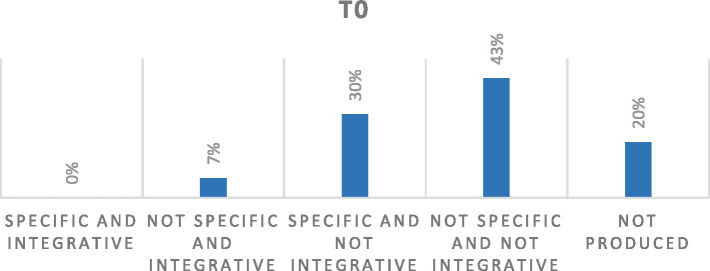
Percentage distribution of quality of SDMs at T0. *Out of a total of 33 texts.

Finally, no memory shows characteristics of specificity and integration (Figure 1).

The memories produced by 11 women at T1, after participating in the group intervention, show how the production of not fully articulated memories by women increases to 44%, probably due to the group process that re-connected and re-actualized in the here-and-now contact with their own cancer story working on the changes introduced on the body level. The work within the group intervention may explain an inhibition of the retrieval process and an emotional avoidance of memories connected to one’s cancer journey.

In total, 30% of memories showed characteristics of non-specificity and non-integration of meaning.

*Waiting. as I wrote above, I am now waiting for a new surgery. And it's a very tiring wait, because it's long and because it forces me to keep open a door that I wanted to start closing. More tiring because you think about doubts: what if the same problem recurs after surgery? What happens if the situation does not improve after the operation? In short, it is a wait full of anxiety. Kept under control, but it's there. (Id 7, 47 years old, year of diagnosis 2019).*

As shown by the literature, the anxiety, named in the narration of the woman’s memory, blocks access to the specificity of the memory and the recovery of greater details.

In total, 20% of the memories show qualities of specificity, with a high negative emotional impact details re-actualized through the narrative in the relationship between here and now and there and then, but with the absence of a process of integration of meaning.

*An unwelcome guest during pandemic. It was 4 June 2020 when I felt a ball in my chest. Immediately I felt it was bad news. I felt it. I was alerted because my aunt had died of breast cancer when I was 16. I immediately called my mother who is a doctor, I did the ultrasound which confirmed the suspicion, and then the MRI. I clearly remember the sensations I felt, I still relive them now: Anguish and despair (Id 2, 47 years old, year of diagnosis 2021).*

**Figure 2:**
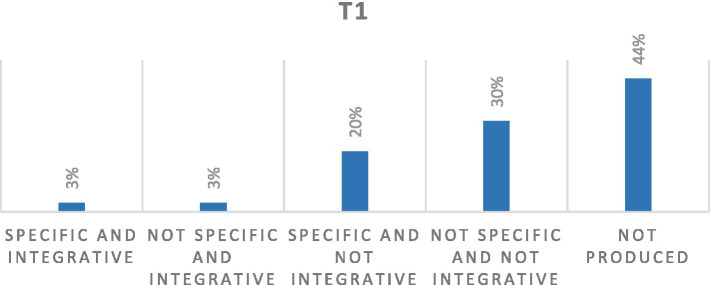
Percentage distribution of quality of SDMs at T1. *Out of a total of 33 texts.

Unlike previous detection (T0), during T1, 3% of memories acquire qualities of both specificity and positive integration of meaning for one’s self and one’s future life trajectory. This can be a result of participation in the group intervention (Figure 2).

*I was there convinced that everything would be fine, and then at the end of the evening the diagnosis, the fear and disbelief in the surgeon's eyes… I thought it would be the end. I will die in excruciating pain, and instead, I am here miraculously, strong, and maybe even better. I understand that I am no longer the same, but if I believe it, something wonderful can still exist for me (Id 5, 57 years old, year of diagnosis 2018).*

The memories produced by 11 women at T2, after 1 month follow the participating in the group intervention, show an increase to 51% of not fully realized memories. Furthermore, as in the T1, women do not retrieve and share the memories of their cancer journey and seem to have increased their inhibition and emotional avoidance, making it a complex internal process and probably excessively demanding for the women themselves once the group intervention has concluded. This seems to attest to how remembering the cancer experience still represents an effort and a painful emotional commitment for women, not only due to the treatments already undergone but also due to the uncertainty toward the future as observable below.

**Figure 3:**
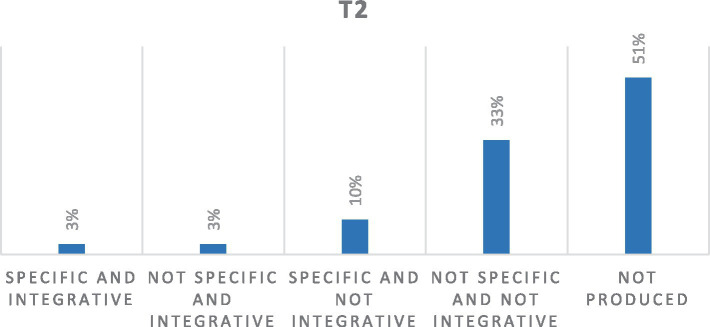
Percentage distribution of quality of SDMs at T2. *Out of a total of 33 texts.

It appears interesting to observe how openness to the process of specificity and the integration of meaning through memories remains at a percentage of 3%. The meaning of memory seems to have aspects linked both to the awareness of one’s own strength and the battle already fought but also to fragility and confusion due to the uncertainty of the future (Figure 3).

*The look. I look at myself without problems, practically immediately after the surgery. I know of other women who can't do it. I look at myself and I look at this further dissimilarity. Also because, okay, the cancer is gone, but I'm waiting for another surgery for a problem with the prosthesis. I look at myself and say, okay, let's move on. I realized that I am strong but I also feel lost and discouraged. I fear that the situation will not improve or that the problem will return … (Id 5, 57 years old, year of diagnosis 2018).*

## Discussion

This study, although in a preliminary way, allowed us to shed light on the process of identity integration of the onset and treatment in women with extended survival from breast cancer through the analysis of their SDMs. Following our exploratory questions, our analysis of the themes of the SDMs highlights the cues and concerns that remain important and salient within the AM of women 7 years after the diagnosis. The three emerging themes were as follows: *The onset of breast cancer*; *The labeling of negative emotions*; and *bodily changes*. In addition, the analysis of the specificity and integration of meaning in memories has allowed us to highlight some clinical reflections regarding how focusing on these dimensions might support memory rescriptiong interventions with women who survived breast cancer.

Regarding the themes that emerged in the first analysis, we found several aspects that confirm the literature in the field. Narratives highlighted the unexpected and shattering experience of the onset of diagnosis. The discovery of cancer is vividly imprinted in one’s memories, often occurring by an accidental discovery, which leaves one disoriented, helpless, and full of terror of death in the face of the interruption of self-continuity opening to catastrophic scenarios for example, recurrent frightening imagery (Laranjeira, 2013; Hendrick et al., 2019) as well as a condition comparable with anticipatory mourning (Martino et al., 2022). Furthermore, the onset of breast cancer encompasses memories connected to the quest for meaning linked to the internal demand, “Why me?” (Gall and Bilodeau, 2017). Often this search takes on the trajectory of recounting a family history that contains a script whose ending appears already written or the search for a sense of control of events by constructing subjective speculations, potential contributors, and predictability around the onset of the cancer (Martino et al., 2023). The issue of bodily change is organized around the loss, the difficulty of recognizing internal and external changes, and the emphasis on the negative sensations and pain that come from the body. The body is remembered as an ambivalent object, which, on the one hand, provides frequent signs of resilience and, on the other hand, can no longer be trusted and could betray one at any time.

Regarding the second analysis involving the specificity and integration of SDMs, over the three time periods, it gives us the opportunity to reflect on the forms of meaning-making with regard to self-understanding, relationship to illness, and experiences of social interaction that might be present in the breast cancer-linked SDMs of these survivors. Furthermore, we were able to examine how the group intervention with its focus on the survivors’ SDMs affected the integrative meanings they assign to their breast cancer-related memories.

SDMs appear inhibited with regard to the specificity of the events remembered (the process of retrieval and recollection of specific episodes). As shown by the literature, anxiety, evident in the narration of the woman’s memory, may have blocked access to the specificity of the memory and the recovery of greater details (Morel et al., 2015). This reduced specificity could potentially be due to defensive mechanisms of emotional avoidance, anxiety, and suppression from contact with the still painful experience, resulting in a progressive failure in recollection/production of detailed imagistic memories. In line with this supposition, Hulbert and Anderson (2018) demonstrated that individuals with a traumatic history showed more suppression-induced forgetting of negative memories compared to the control group. Particularly, the authors reported a process of a greater generalized forgetting of suppressed materials in participants. Thus, it would be valuable to foster a cognitive reconstruction and a retrospective re-evaluation to reframe past experiences with a focus on more redemptive meaning-making (Baker et al., 2017; Samide and Ritchey, 2021). Similarly, finding a “silver lining” (Holland and Kensinger, 2013) can reduce the distress and negative emotions that afflict the subject during a critical event and in remembering the memory (see the *reconsolidation process*, Hallford et al., 2024).

The present findings suggest that a major aspect of difficulty for these survivors is drawing from their memories an integrative and constructive meaning for their future life or for their relationship with the world/others. The cancer journey experience seems to remain separated from their own identity and autobiographical story and therefore not yet usable to extract an integrative meaning.

On a more immediate clinical level, the psychological work within the support group may explain an inhibition of the retrieval process linked to the re-actualization of the pain story during the surgery and treatment and the salience of bodily changes, opening an emotional avoidance of memories connected to one’s cancer journey. In other words, the power of the group expression may also instigate some defensive avoidance on the part of the group members. Clinicians working with survivors would benefit from moving carefully into these most vulnerable areas, building up trust and mutual support in the participants to allow for more open self-disclosure.

On a more specific level, it seems interesting to discuss how 7% of memories, although they do not show specific characteristics, access a process of integration of negative and devaluing meaning-making for the self and one’s relationship with future life and one’s world view. On the contrary, it appears interesting to observe how openness to the process of specificity and the integration of meaning through memories remains, in the T1 and T2, at a percentage of 3%. For this minority within the group, the meaning of memory seems to have aspects linked both to the awareness of one’s own strength and to the battle already fought. Despite the fragility and confusion due to the uncertainty of the future, these memories reflect a trust in more hopeful outcomes that will arrive in the future. This can be a result of the participation within the group intervention, whereas for some it represented a space to broaden the understanding of their experience of illness by integrating new meanings about themselves and their future. It will be important to explore in depth the psychological functioning (symptoms, level of processing, etc.) of those women who most benefited from the group support.

We believe, in conclusion, that our results, in accordance with the literature, highlight how people with experience of cancer show a renegotiation of self over time. Specifically, changes in biography across cancer are generally focused on four core components, as follows: the patient’s inner world, in terms of psychological issues, emotions, and coping skills; the embodied self, involving the body-and-mind interconnections, self in the relationship with other, and self in reference to the context to their place in the world and larger society. All these themes are in a constant interrelationship over time, which is fundamental to identity renegotiation and adaptation (Pearce et al., 2020). Therefore, managing memories is essential throughout the healthcare process to promote better identity adaptation and for the navigation of effective coping and emotional regulation (Jahnen et al., 2021).

For a small, but meaningful subset of the survivor group, thanks to the reflection with women who have gone through the same illness experience, they built a transformative space to cope with the cancer experience, both in the immediacy of the group, but also in a more extended restructuring of their self-defining memories. They expanded their breast cancer memories’ meaning to go beyond negative emotions and recast them in a more constructive and optimistic light.

Breast cancer has remained an ongoing experience for all of these survivors, persisting into the present moment, even 7 years after diagnosis. For most women, its continued highly negative emotional impact may block the possibility of constructing a more adaptive meaning from one’s cancer memories, thus integrating them into one’s identity and autobiographical life experience in order to sustain a healthy psychological recovery (see, Nieto et al., 2019).

## Limitations and conclusion

From a clinical point of view, this study suggests the importance, to be confirmed in future studies, of clinical support focused on the AM in long survivorship for breast cancer and centralized on the cognitive reconstruction and reframing of past memories. Such memory work could affect how the experience is stored in one’s identity, creating spaces for access to the transformation of the meaning of the remembered experience. Although the present study has several limitations including the low sample size, the absence of a control group, and external and concurrent measures such as depression or anxiety scales, we believe that this study opens up useful reflections from a clinical point of view to support breast cancer long survivorship in women. We encourage future studies to engage with longitudinal design research and enlarge the study of AM functioning with other kinds of cancer. In addition, future studies could include a collection of independent behavioral data that might provide validation of the participants’ subjective reports, while also avoiding the influence of social desirability effects related to the clinical intervention.

## Data Availability

The raw data supporting the conclusions of this article will be made available by the authors, without undue reservation.
